# The advanced performance of microbial consortium for simultaneous utilization of glucose and xylose to produce lactic acid directly from dilute sulfuric acid pretreated corn stover

**DOI:** 10.1186/s13068-021-02085-8

**Published:** 2021-12-07

**Authors:** Yaqin Sun, Xiaoying Li, Lida Wu, Yi Li, Fan Li, Zhilong Xiu, Yi Tong

**Affiliations:** 1 School of Bioengineering, Dalian University of Technology, No. 2 Linggong Road, Ganjingzi District, Dalian City, Liaoning Province 116024 People’s Republic of China; 2 COFCO Biochemistry Co., Ltd. (National Engineering Research Center of Corn Deep Processing), Changchun City, Jilin Province 130033 People’s Republic of China

**Keywords:** Lactic acid, Microbial consortium, Corn stover, Simultaneous saccharification and co-fermentation (SSCF), Non-detoxification

## Abstract

**Background:**

Lignocellulosic feedstocks have attracted much attention as a potential carbon source for lactic acid (LA) production because of their ready availability, sustainability, and renewability. However, there are at least two major technical challenges to producing LA from lignocellulose. Inhibitors derived from lignocellulose pretreatment have a negative impact on the growth of cells producing LA. Furthermore, pentose sugars produced from the pretreatment are difficultly utilized by most LA producers, which is known as the carbon catabolite repression (CCR) effect. This complex feedstock can be utilized by a robust microbial consortium with high bioconversion efficiency.

**Results:**

In this study, a thermophilic consortium DUT50 producing LA was enriched and employed to improve corn stover (CS) utilization. *Enterococcus* was the dominant family in the consortium DUT50, accounting for 93.66% of the total abundance, with *Lactobacillus*, *Bacillus*, *Lactococcus*, and *Trichococcus* accounted for the remaining 2.68%. This consortium could be resistant to inhibitors concentration up to 9.74 g/L (2.88 g/L acetic acid, 2.46 g/L furfural, 2.20 g/L 5-HMF, and 2.20 g/L vanillin derived from pretreatment of CS), and simultaneously metabolizes hexose and pentose without CCR effect. Based on the promising consortium features, an efficient process of simultaneous saccharification and co-fermentation (SSCF) was developed to produce LA from acid pretreated corn stover, in which solid–liquid separation and detoxification were avoided. The key influencing factors were investigated and optimized, including dry biomass and cellulase loading, corn steep liquor powder concentration, and the pre-hydrolysis time. The highest LA titer of 71.04 g/L with a yield of 0.49 g/g-CS was achieved at a dry biomass loading of 20% (w/v), which is the highest LA production from non-detoxified acid pretreated corn stover via the SSCF process without wastewater generation reported to date. The simultaneous metabolism of hexose and pentose revealed collaboration between *Enterococcus* in the consortium, whereas xylose may be efficiently metabolized by *Lactobacillus* and *Bacillus* with low abundance via the pentose phosphate pathway.

**Conclusions:**

The experimental results demonstrated the potential advantage of symbiosis in microbial consortia used for LA production from lignocellulosic biomass.

## Background

Lactic acid (LA), an important chemical, has a wide range of applications in the food, pharmaceutical, and chemical industries, particularly as a monomer of biodegradable and biocompatible polylactic acid [1, 2]. Low-cost lignocellulosic feedstocks have received a lot of attention as an alternative carbon source for LA production with ready availability, sustainability, and renewability [3, 4]. However, the LA production from lignocellulose faces at least two major technical obstacles. Pretreatment of lignocellulosic biomass was one of the obstacles that hindered lignocelluloses utilization due to its recalcitrant nature [5]. The formation of organic acid and furan derivatives during the lignocellulose pretreatment may inhibit microbial growth and subsequent fermentation [6, 7]. Furthermore, lignocellulose hydrolysis yields a mixture of hexose and pentose sugars. However, most LA producers are unable to ferment pentose sugars and exhibit carbon catabolite repression (CCR) effect [8].

Many studies have been conducted to evaluate the degradation of lignocellulosic biomass and the production of LA using physical, chemical, and biological pretreatments. Dilute acid pretreatment was used among these pretreatment methods to efficiently produce lactic acid [7, 9, 10]. A high LA titer of 101.9 g/L was obtained from sulfuric acid pretreated and bio-detoxified corn stover (CS). The poor yield of a mere 0.38 g/g stover was due to *P. acidilactici* DQ2 not utilizing xylose. Although *P. acidilactici* DQ2 showed tolerance to lignocellulose-derived inhibitors, a bio-detoxification procedure using fungus of *A. resinae* ZN1 was applied to pretreated CS for five days until 90% of furfural and hydroxymethyfurfural (HMF) were removed. L-LA was also produced by engineered *Pediococcus acidilactici* TY112 with *ldh* gene disruption from dilute acid pretreated and bio-detoxified corn stover feedstock [9]. A LA titer of 77.66 g/L was achieved at 25% (w/w) solids content. An efficient process from sulfuric acid pretreated wheat straw without solid–liquid separation and detoxification was achieved by *B. coagulans* IPE22 [6]. As a result, 38.73 g/L-LA was obtained with a yield of 0.46 g/g-WS. The obtained titer, however, was insufficient to compete with commercial LA production. The results indicated that the level of inhibitors derived from lignocellulose and utilization efficiency of pentose have a strong influence on lactic acid titer, yield, and productivity using lignocellulose as feedstock.

According to published research, dilute acid pretreatment increases cellulose availability by solubilizing hemicellulose into pentose sugars. However, lignocellulose-derived inhibitors have a significant impact on LA production. Following pretreatment, water washing for pH adjustment or complex detoxification procedures could remove the aforementioned inhibitory compounds, but this process will inevitably result in hemicellulose loss, a large amount of industrial wastewater, and an increase in the environmental burden [3, 7, 11]. Furthermore, the low efficiency of pentose utilization and CCR effect result in low LA productivity, making LA production from lignocellulosic feedstocks less sustainable and cost competitive. As a result, strains with the remarkable capabilities to co-ferment pentose/hexose efficiently while also being resistant to inhibitors were desired and preferred.

Currently, carbohydrate fermentation using pure cultures of lactic acid bacteria is used in almost all commercial lactic acid production [12]. Compared to pure culture fermentation, microbial consortium shows outstanding robustness against environmental disturbances and microbial invasion. Especially, microbial consortium can use renewable and complex feedstocks displaying a higher bioconversion efficiency than pure culture, requiring less expensive purification processes [13]. Recently, microbial consortium has been used to produce LA from un-pretreated sugarcane molasses [14] and hydrolysate of corn stover [15, 16]. The results showed that microbial consortium could overcome a high metabolic burden bottleneck and inhibition of the toxic byproducts derived from the biomass pretreated process. However, the effect of inhibitors on microbial consortium and the bioconversion process as well as the microbial consortium’s interaction mechanism has been little studied and remains unknown.

This study’s objective was to evaluate the feasibility of lactic acid production from acid pretreated corn stover (pre-CS) without detoxification via simultaneous saccharification and co-fermentation (SSCF) by thermophilic microbial consortium. To meet the optimal hydrolysis temperature of cellulase, a robust microbial consortium with high thermotolerance up to 50 °C was enriched and adapted using an adaptive evolution engineering strategy. To emphasize, a feasible route without solid–liquid separation and detoxification was investigated by the microbial consortium to reduce wastewater generation and improve LA production. The impact of total inhibitors derived from acid pretreatment on the microbial consortium DUT50 was investigated. Furthermore, the key influencing factors for the SSCF process, such as cellulase loading, dry biomass loading, corn steep liquor powder (CSLP) loading, and the pre-hydrolysis time, were optimized. Finally, the interaction mechanism of microbial consortium DUT50 was investigated through serial dilution and construction of the synthetic microbial consortium.

## Results and discussion

### Sugars and inhibitors liberated during pretreatment of CS by dilute H_2_SO_4_

The untreated corn stover (% w/w, on a dry basis) was primarily composed of cellulose (31.18 ± 0.23), hemicellulose (26.95 ± 0.14), and lignin (16.43 ± 0.27). The CS used in this study contained less cellulose, while hemicellulose and lignin levels were comparable to previous studies [5, 10]. Differences in the components of CS were most likely caused by trait variations and growing lands. In this study, CS was first pretreated with dilute H_2_SO_4_ solution before releasing reducing sugars and the inhibitors. The effects of different CS loading on sugars and inhibitory conversion were determined via diluted-H_2_SO_4_ pretreatment. The concentrations of liberated sugars and inhibitors in the hydrolysate liquors from various CS loadings are shown in Table 1. Xylose was presented as the major sugar produced in the hydrolysate liquors, followed by glucose and arabinose. At a dry biomass loading of 35% (w/v), a high xylose concentration of 45.22 g/L was obtained, along with a glucose concentration of 8.30 g/L and an arabinose concentration of 6.07 g/L. It could be because hemicellulose has a lower molecular weight, is less lignified, and is more amorphous than cellulose. Thus, it is easily hydrolyzed by acids [17]. The percentages of hemicellulose saccharification and cellulose saccharification decreased as the increase of corn stover loading during dilute acid pretreatment. The generated inhibitors, including organic acid (acetic acid) and furan derivatives (furfural, 5-hydroxymethyl furfural, and vanillin), clearly increased with increasing CS loading. When the dry biomass loading ranged from 5% (w/v) to 35% (w/v), the total inhibitors increased from 5.37 g/L to 12.59 g/L. The rice straw pretreated with H_3_PO_4_ showed significantly lower total inhibitors when compared to those of the pretreatments with H_2_SO_4_ and HCl [17]. The total inhibitors of 5.10 g/L were obtained when 1 N H_2_SO_4_ pretreated rice straw with a dry biomass loading of 10% (w/v) for 60 min. Total inhibitors of 6.99 g/L were obtained when 1% (v/v) H_2_SO_4_ pretreated CS with a dry biomass loading of 10% (w/v) for 120 min, as shown in Table 1. In this study, the lignin content of CS (16.43%, w/w) was comparable to that of rice straw (15.10%, w/w). It may also imply that pretreatment by a weak acid with a longer reaction time is preferred to obtain more undesirable byproducts.

**Table 1:** The components of H_2_SO_4_ pretreated CS hydrolysate liquors with various dry biomass loadings

Dry loading (%, w/v)	Sugar concentration (g/L)			Inhibitors concentration (g/L)			
	Glucose	Xylose	Arabinose	Acetic acid	Furfural	5-HMF	Vanillin
5	1.13 ± 0.10	8.44 ± 0.04	1.22 ± 0.02	0.93 ± 0.03	1.46 ± 0.01	1.35 ± 0.01	1.63 ± 0.01
10	3.49 ± 0.10	17.59 ± 0.08	1.35 ± 0.04	1.75 ± 0.01	1.82 ± 0.02	1.49 ± 0.02	1.93 ± 0.03
15	4.12 ± 0.30	23.64 ± 0.78	4.32 ± 0.85	2.51 ± 0.01	2.18 ± 0.02	1.93 ± 0.01	2.00 ± 0.03
20	6.14 ± 0.42	29.17 ± 0.77	3.28 ± 0.42	2.88 ± 0.04	2.46 ± 0.14	2.20 ± 0.08	2.20 ± 0.05
25	6.80 ± 0.18	33.97 ± 0.96	4.44 ± 0.22	3.24 ± 0.37	2.92 ± 0.06	2.45 ± 0.05	2.24 ± 0.01
30	7.71 ± 0.13	38.69 ± 0.07	5.50 ± 0.08	4.28 ± 0.05	2.96 ± 0.03	2.82 ± 0.05	2.25 ± 0.02
35	8.30 ± 0.43	45.22 ± 0.02	6.07 ± 0.38	4.43 ± 0.60	2.98 ± 0.03	2.97 ± 0.01	2.28 ± 0.01

### Adaptive evolution of microbial consortia to high temperature and inhibitors

To ensure efficient lactic acid production from agricultural waste substrate, a robust microbial consortium with a wide range of substrate utilization capability and resistance to different inhibitors or stressful conditions is required. In this study, to meet optimal hydrolysis temperature of cellulase and have high resistance to inhibitors, a microbial consortium was enriched and adapted via adaptive evolution strategy of gradually increasing temperature from 42 °C to 50 °C at a high inhibitors concentration of 6.99 g/L. Long-term domestication in pre-CS with non-detoxified hydrolysate liquor resulted in a stable and functional microbial consortium with heat-resistant and inhibitor-tolerant capacities. 16S rRNA gene amplicon high-throughput sequencing was performed to investigate the bacterial composition of the microbial consortium DUT50 during the evolution process. The result is presented in Table 2. *Escherichia-Shigella* was the predominant family at cultivation temperatures of 42 °C and 45 °C, accounting for 95.30% and 82.80%, respectively. Under the same conditions, *Enterococcus* had a lower proportion. Only 4.18% abundance was detected at 42 °C. With increasing cultivation temperature, *Enterococcus* became the dominant family at 47 °C and 50 °C, accounting for 90.82% and 93.66%, respectively. At 50 °C, no *Escherichia-Shigella* was found in the consortium DUT50. At the same level of inhibitors, the abundance analysis revealed that the genus of *Enterococcus* is more resistant to high temperatures than the genus of *Escherichia-Shigella*. Moreover, the abundance of *Lactobacillus*, *Bacillus*, *Lactococcus*, and *Trichococcus*, accounting for 2.68% in total, increased at 50 °C. *Lactococcus* and *Trichococcus*, in particular, did not exist in the microbial consortium when cultivation temperature was lower than 47 °C. This result suggested that *Lactococcus* and *Trichococcus* have a high heat-resistant capacity.

**Table 2:** Microbial community analysis of the adaptive consortia and the diluted consortia of DUT50

Taxonomy	Percentage (%)							
	DUT42 (42 °C) ^a^SRR14149087	DUT45 (45 °C) ^a^SRR14149086	DUT47 (47 °C) ^a^SRR14149085	DUT50 (50 °C) ^a^SRR14149088	Diluted consortia of DUT50			
					× 10^–2^ ^a^SRR14149131	× 10^–4^ ^a^SRR14149130	× 10^–6^ ^a^SRR14149129	× 10^–8^ ^a^SRR14149128
*Escherichia-Shigella*	95.30	82.80	0.04	–	–	–	–	–
*Enterococcus*	4.18	16.43	90.82	93.66	99.27	99.53	98.89	99.62
*Lactobacillus*	0.08	0.12	0.60	1.05	–	–	–	–
*Bacillus*	0.05	0.04	0.18	0.87	–	–	–	–
*Lactococcus*	–	–	–	0.38	–	–	–	–
*Trichococcus*				0.38	–	–	–	–
*Burkholderia*	0.002	–	0.04	–	0.22	0.13	0.42	0.07
*Streptophyta*				0.02	0.02	0.01	0.02	–
*Unclassified*	0.12	0.30	3.33	3.48	0.35	0.19	0.29	0.28

^a^The accession number of microbial consortia in NCBI Sequence Read Archive

These unique thermophilic and inhibitors-tolerant properties of microbial consortium DUT50 may benefit the SSCF process of acid pretreated CS without solid–liquid separation and detoxification, which matches the optimal cellulase activity, reduces microbial contamination risks, and has a high tolerance to inhibitors derived from the pretreatment process.

### Lactic acid production directly from pretreated corn stover by microbial consortium

#### Fermentation of hydrolysate liquor

**Fig. 1:**
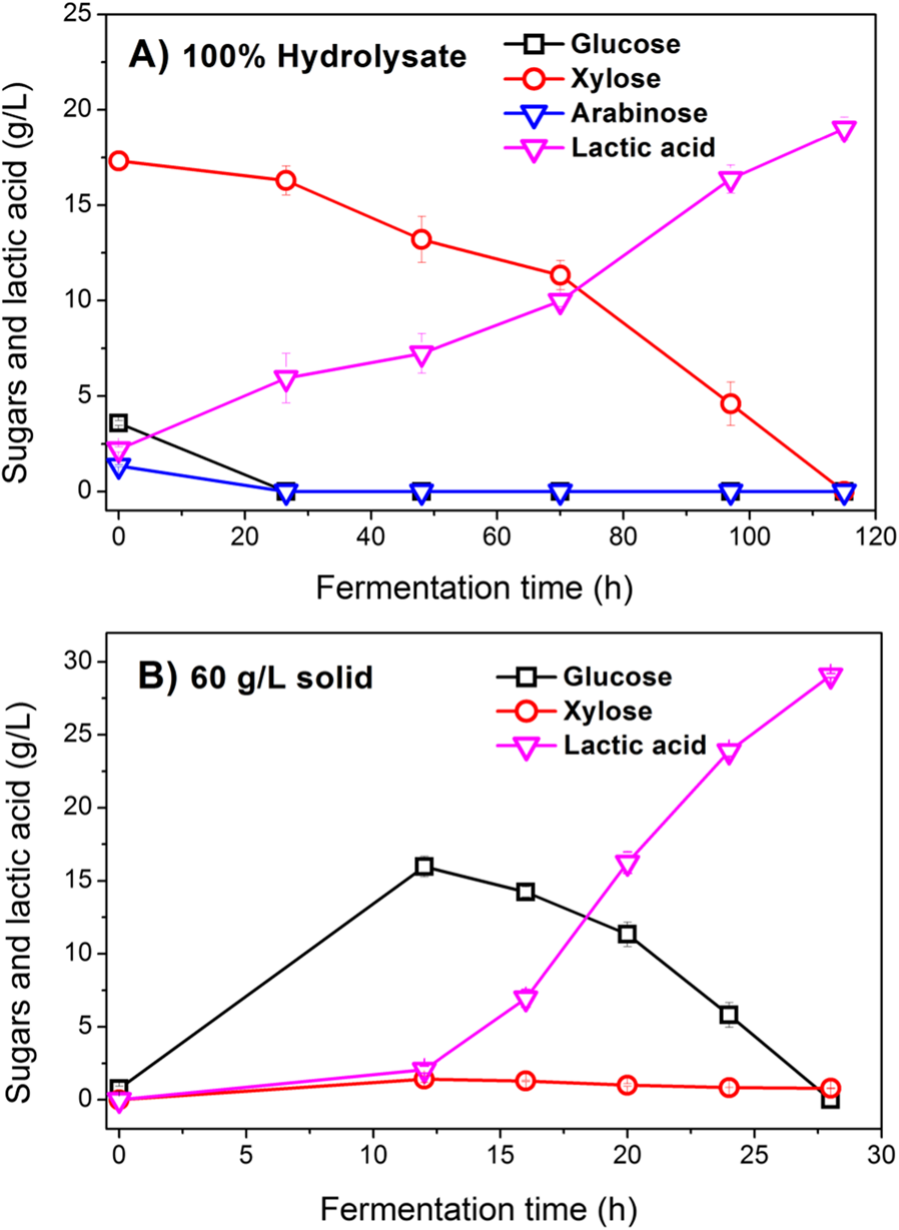
Lactic acid production by consortium DUT50 from the hydrolysate liquor and solid fraction of pre-CS. **A** 100% (v/v) hydrolysate liquor. **B** 60 g/L solid fraction. Conditions: 1 L bioreactor containing 500 mL CSLP medium, 10% (w/v) of dry biomass loading, 25 FPU/g-CS cellulase, 20 g/L CSLP, 12 h pre-hydrolysis time, 50 °C, pH 5.5

At the dry CS loading of 10% (w/v), the total sugars and the total inhibitors in the hydrolysate liquor of pre-CS were approximately 22.24 g/L and 6.99 g/L, respectively. The total inhibitors included 1.75 g/L acetic acid, 1.82 g/L furfural, 1.49 g/L 5-HMF, and 1.93 g/L vanillin. Microbial consortium DUT50 can grow and produce lactic acid from hydrolysate liquor at this level of total inhibitors (Fig. 1A). This could be because adaptive evolution engineering has improved the inhibitor tolerance of the microbial consortium DUT50. It is critical that the microbial consortium DUT50 be able to use glucose, xylose, and arabinose in the hydrolysate without experiencing CCR effect. DUT50 completely consumed glucose, xylose, and arabinose in 115 h at 6.99 g/L of total inhibitors. Furthermore, lactic acid was produced as the sole product, with no other organic acid detected, indicating that microbial consortium DUT50 may be primarily composed of homo-fermentative lactic acid-producing strains. As a result, a lactic acid concentration of 19.01 g/L was obtained from 100% (v/v) hydrolysate liquor, with a comparable yield to total reducing sugars of 0.96 g/g. Although the microbial consortium DUT50 can grow in the presence of high concentrations of inhibitors, a weak ability of the consortium DUT50 to metabolize xylose results in a relatively low lactic acid productivity of 0.17 g/(L h) at an average xylose consumption rate of 0.15 g/(L h).

A previous study found that 2 g/L acetic acid inhibited the growth of *Pichia stipitis* for ethanol production [18]. The growth of *C. tyrobutyricum* and butyric acid production were completely inhibited by 1.2 g/L furfural and 2.4 g/L HMF, respectively [19]. It has been reported that *Pediococcus acidilactici* DQ2 has an extraordinary tolerance to 3.0 g/L furfural and 3.0 g/L HMF [7]. However, *P. acidilactici* DQ2 was relatively sensitive to formic acid and vanillin. As a result, 0.5 g/L formic acid and 0.2 g/L vanillin will inhibit both cell growth and lactic acid fermentation performance. During the succinate production, the H_3_PO_4_ pretreated hydrolysate possessed the minimum inhibitory concentration up to 1.84 g/L for *E. coli* AS1600a [17]. In their study, the detoxification by adjusting the hydrolysate to pH 9 by NH_4_OH was adopted to lower the inhibitors toxicity. *B. coagulans* IPE22 showed significant growth inhibition at 1.0 g/L formate, 3.0 g/L furfural, and 2.0 g/L 5-HMF, respectively [6]. Furthermore, IPE22 demonstrated excellent resistance to acetate and vanillin.

CCR-negative strains were preferred for biochemicals production from mixed sugars derived from lignocellulosic biomass. Some strains, such as *E. coli* [17] and *B. coagulans* [6], have been reported to utilize glucose, xylose, and arabinose simultaneously without CCR effect. Engineered *E. coli* AS1600a offers co-transporting glucose and xylose with the same transporter in which the CCR regulation is relieved. As a result, it may efficiently generate ATPs from glycolysis while reserving them for xylose metabolism via the pentose phosphate pathway.

### SSCF of cellulose solid fraction

Hemicellulose and lignin could be effectively dissolved by acid pretreatment. Approximately 40% (w/w) of CS was hydrolyzed through pretreatment, and 60% (w/w) residues were retained at a dry biomass loading of 10% (w/v). For 60 g/L pretreated CS solid fraction with a pre-hydrolysis time of 12 h, the maximum glucose and xylose were obtained (Fig. 1B). Consortium DUT50 used xylose and glucose simultaneously and completely consumed them in 28 h. At the end of the fermentation, a lactic acid concentration of 29.06 g/L was produced with a yield to solid of pre-CS of 0.56 g/g. Lactic acid productivity via the SSCF process was found to be higher at 60 g/L pretreated CS solid fraction than at 100% (v/v) hydrolysate fraction. The pretreated CS solid fraction had a lactic acid productivity of 1.04 g/(L h), which was 593% higher than the hydrolysate fraction (0.15 g/(L h)). It is due to the low xylose utilization rate and the toxicity of inhibitors in hydrolysate liquor. The hemicellulose degradation during the acidic thermochemical pretreatment produced the majority of xylose in the hydrolysate fraction, but the pretreated CS solid fraction produced a relatively low amount of xylose (only 1.42 g/L) after enzymatic hydrolysis. Water washing was also used to remove the inhibitors from the CS solid fraction. As a result, lactic acid productivity was relatively high.

In general, the washing process was applied to remove inhibitors that remained in the solid fraction and to adjust the pH to neutral. In fact, the washing-based detoxification and neutralization process would inevitably generate a large amount of wastewater and increase operating costs. In this study, the operation of washing detoxification was omitted, and the solid fraction was directly utilized to produce lactic acid. Due to the low dry biomass loading, a comparable yield of 0.56 g/g to solid was obtained, and the obtained concentration was not competitive.

### SSCF of acid pretreated corn stover

**Fig. 2:**
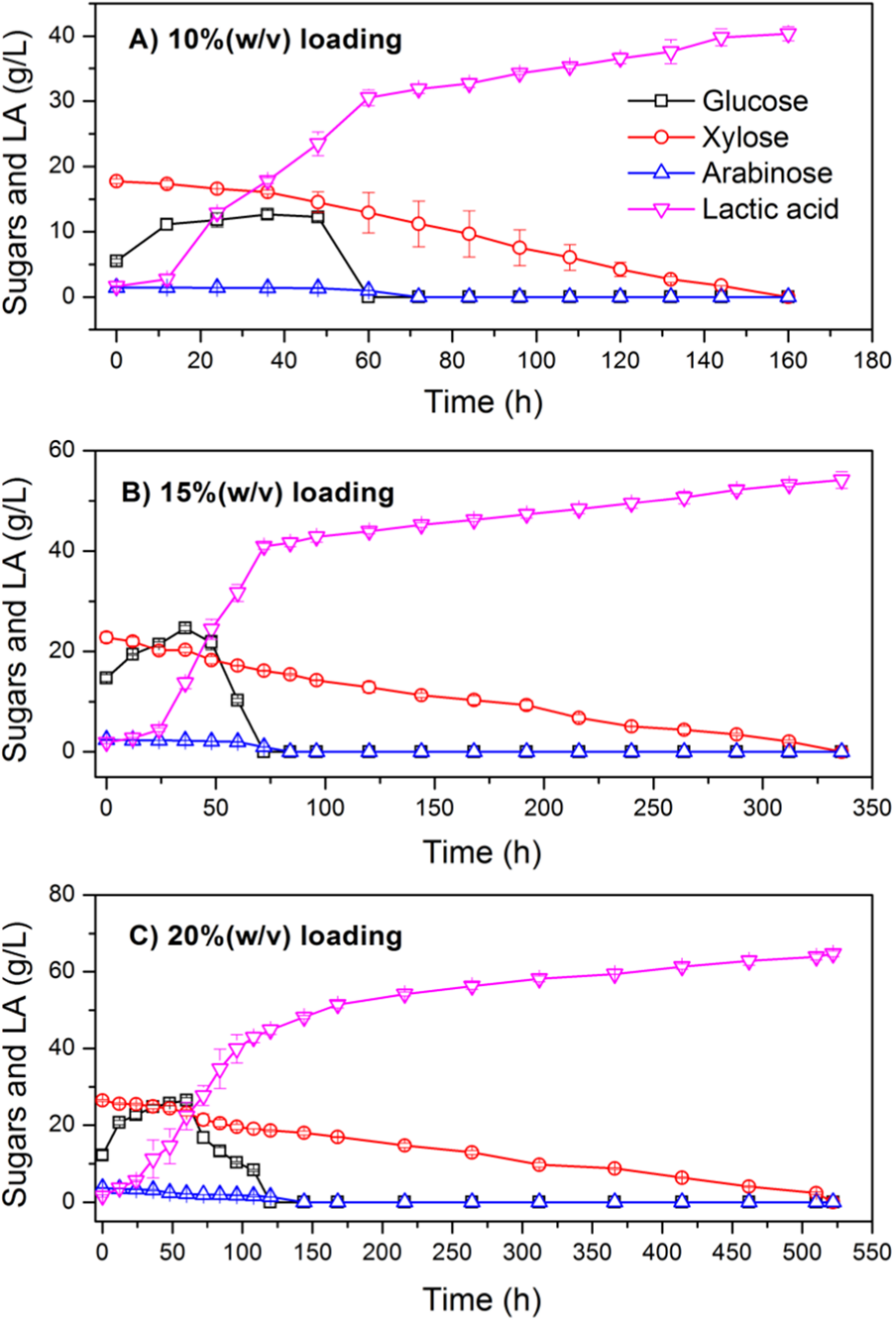
LA production via the SSCF process from non-detoxified acid pretreated corn stover by microbial consortium DUT50. **A** 10% (w/v) dry biomass loading, 6.99 g/L total inhibitors. **B** 15% (w/v) dry biomass loading, 8.62 g/L total inhibitors; (**C**) 20% (w/v) dry biomass loading, 9.74 g/L total inhibitors. Conditions: 1 L bioreactor containing 500 mL CSLP medium, 35 FPU/g-CS cellulase, 20 g/L CSLP, 12 h pre-hydrolysis time, 50 °C, pH 5.5

Actually, it was proposed to use both hemicellulose hydrolysate liquor and cellulose solid fraction derived from lignocellulosic biomass to produce more sustainable productions of biofuels and biochemicals while reducing waste liquid generation. As a result, the SSCF of pretreated CS, including hydrolysate liquor and solid for lactic acid production, was investigated in this study. The effect of dry biomass loading from 10% (w/v) to 20% (w/v) and total inhibitors concentration from 6.99 g/L to 9.74 g/L on LA production was evaluated by microbial consortium DUT50. Figure 2 shows LA production by the microbial consortium DUT50 via the SSCF process from non-detoxified acid pretreated CS at different dry biomass loadings. A similar pattern of sugar utilization and lactic acid production was observed. Microbial consortium DUT50 grew and produced lactic acid from non-detoxified hydrolysate of CS and showed significant tolerance to total inhibitors up to 9.74 g/L. The total inhibitors included 2.88 g/L acetic acid, 2.46 g/L furfural, 2.20 g/L 5-HMF, and 2.20 g/L vanillin. The high total inhibitors concentration had little effect on the consortia growth or the consumption of xylose and glucose. This is the highest inhibitors concentration reported to be capable of the cell growth for LA production.

Most microorganisms could not metabolize xylose and glucose at the same time, and the xylose consumption began only when almost no glucose remained in the medium. Our research also discovered that xylose and arabinose could be consumed alongside glucose without CCR. The average consumption rate of xylose in media containing the mixture of glucose, xylose, and arabinose was 0.12 g/(L h) at the total inhibitors of 6.99 g/L. In the presence of glucose, the microbial consortium DUT50 consumed xylose slowly. The reason might be that the expression of xylose catabolizing genes in DUT50 was less repressed by glucose than those in other bacteria [5]. Lactic acid concentration increased along with the dry biomass loading and total inhibitors. Moreover, a maximum lactic acid titer of 64.64 g/L with a yield of 0.45 g/g-CS was produced at the dry biomass loading of 20% (w/v) and total inhibitors of 9.74 g/L. LA yield to CS decreased when the total inhibitors and dry biomass loading increased. As a result, a yield of 0.50 g/g-CS was obtained at a dry biomass loading of 10% (w/v) and a total inhibitors concentration of 6.99 g/L. It might contribute to the high efficiency of heat and mass transfers with the decreasing dry biomass loading. In our previous study, different microbial consortia were enriched at different temperatures, and their lactic acid production abilities were compared [16]. The final concentration of lactic acid of 43.73 g/L, with a yield of 0.50 g/g-CS and average productivity of 0.32 g/(L h), was obtained at 10% (w/v) dry CS loading and SSCF temperature of 47 °C. The results showed that microbial consortium exhibited similar properties for lactic acid production via the SSCF process at different temperatures.

Despite the high lactic acid concentration obtained using hemicellulose hydrolysate liquor and cellulose solid fraction derived from lignocellulosic biomass, the average lactic acid productivity was low, with only 0.12 g/(L h) obtained at a dry biomass loading of 20% (w/v), due to a low xylose consumption rate at high inhibitor concentrations. In the development of a synthetic microbial consortium, more emphasis should be placed on the improvement of xylose metabolism.

Microbial consortium is not only resistant to inhibitors derived from pretreatment, but it can also use glucose, xylose, and arabinose concurrently CCR effect. The thermophilic microbial consortium DUT50 was characterized, and its potential application for bioconversion of lignocellulosic biomass to lactic acid was demonstrated.

### Optimization of SSCF process

To achieve economic LA production, various influential factors in the SSCF process with microbial consortium DUT50 were investigated. In this study, the cellulase loading, CSLP concentration, and the pre-hydrolysis time were optimized.

**Fig. 3:**
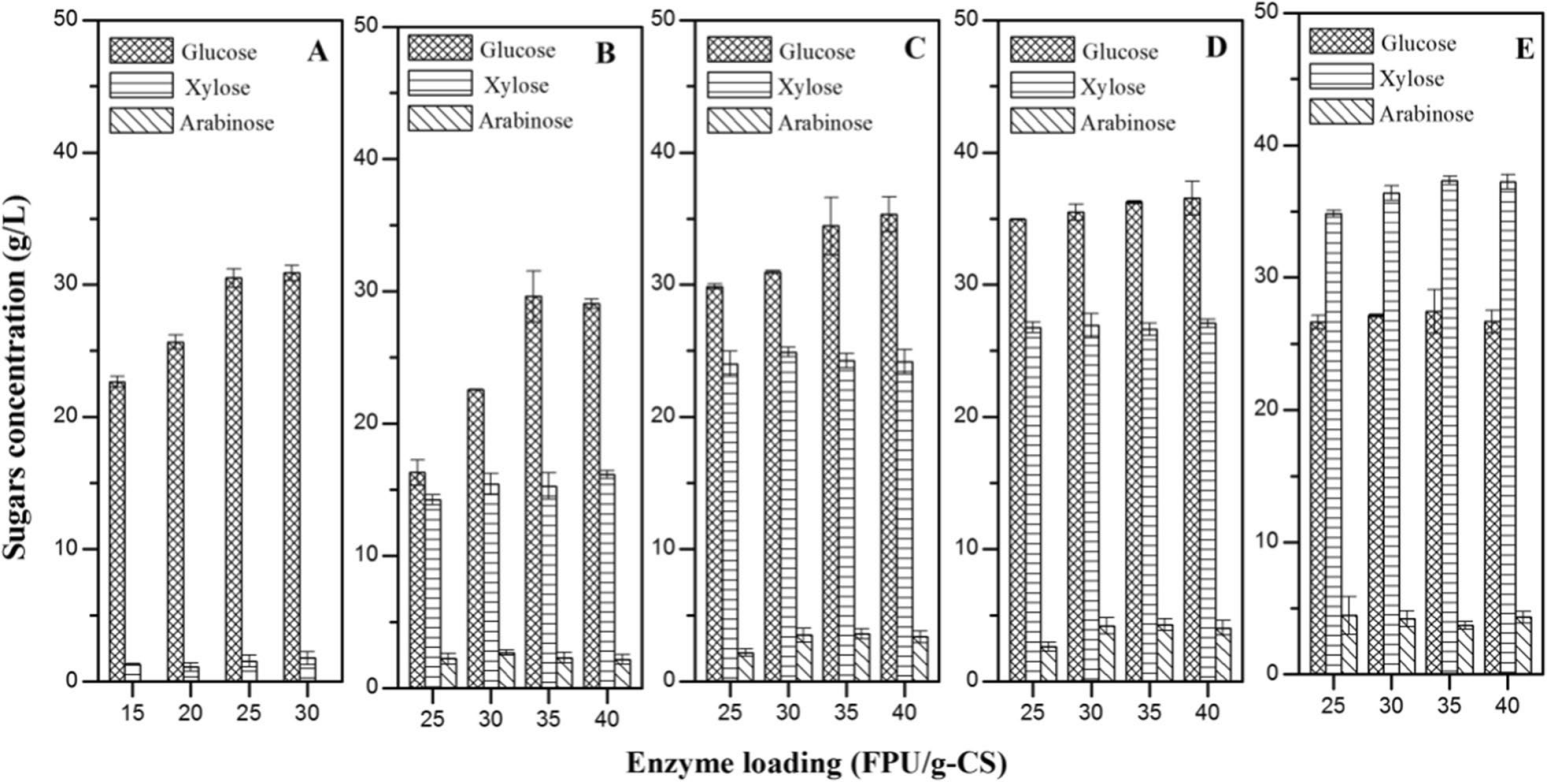
The effect of inhibitors and enzyme loading on the hydrolysis of detoxified and non-detoxified pre-CS. **A** 6% (w/v) solid fraction of pre-CS, 0 g/L total inhibitors. **B** 10% (w/v) dry biomass loading, 6.99 g/L total inhibitors. **C** 15% (w/v) dry biomass loading, 8.62 g/L total inhibitors. **D** 20% (w/v) dry biomass loading, 9.74 g/L total inhibitors. **E** 25% (w/v) dry biomass loading, 10.85 g/L total inhibitors. Conditions: 1 L bioreactor containing 500 mL CSLP medium, 25–40 FPU/g-CS cellulase, 20 g/L CSLP, 50 °C, pH 5.5

In the SSCF process, cellulase loading was critical for saccharification. The inhibitors in hydrolysate liquor have an effect on cellulose saccharification by cellulase, especially when non-detoxified pre-CS in used. The effect of cellulase loading and derived inhibitors from lignocellulose pretreatment on hydrolysis of CS is shown in Fig. 3. The results showed that increasing cellulase loading resulted in higher total sugar concentration. Despite cellulase loading higher than 35 FPU/g-CS, no significant differences were observed for glucose, xylose, and arabinose concentration in non-detoxified CS. When the dry biomass loading was higher than 25% (w/v), the efficiency of enzymatic saccharification was decreased (Fig. 3E). It may be because the excess of enzyme absorbed onto the surface of CS restricted the diffusion process through the cellulose structure. When inhibitors were removed by washing, a higher total sugar concentration for the hydrolysis of solid fraction in pre-CS was obtained under the less cellulase loading (Fig. 3A). The inhibitors derived from the pretreatment of CS have an adverse impact on saccharification by cellulase. The effect could be eliminated via the operation of washing, bio-detoxification, sodium bisulfite addition, etc. [7, 10, 17]. However, washing would generate a large quantity of wastewater, whereas bio-detoxification would take a long time. In addition, the hydrolysate fraction of pre-CS was also wasted, lowering the utilization yield of the total corn stover.

**Fig. 4:**
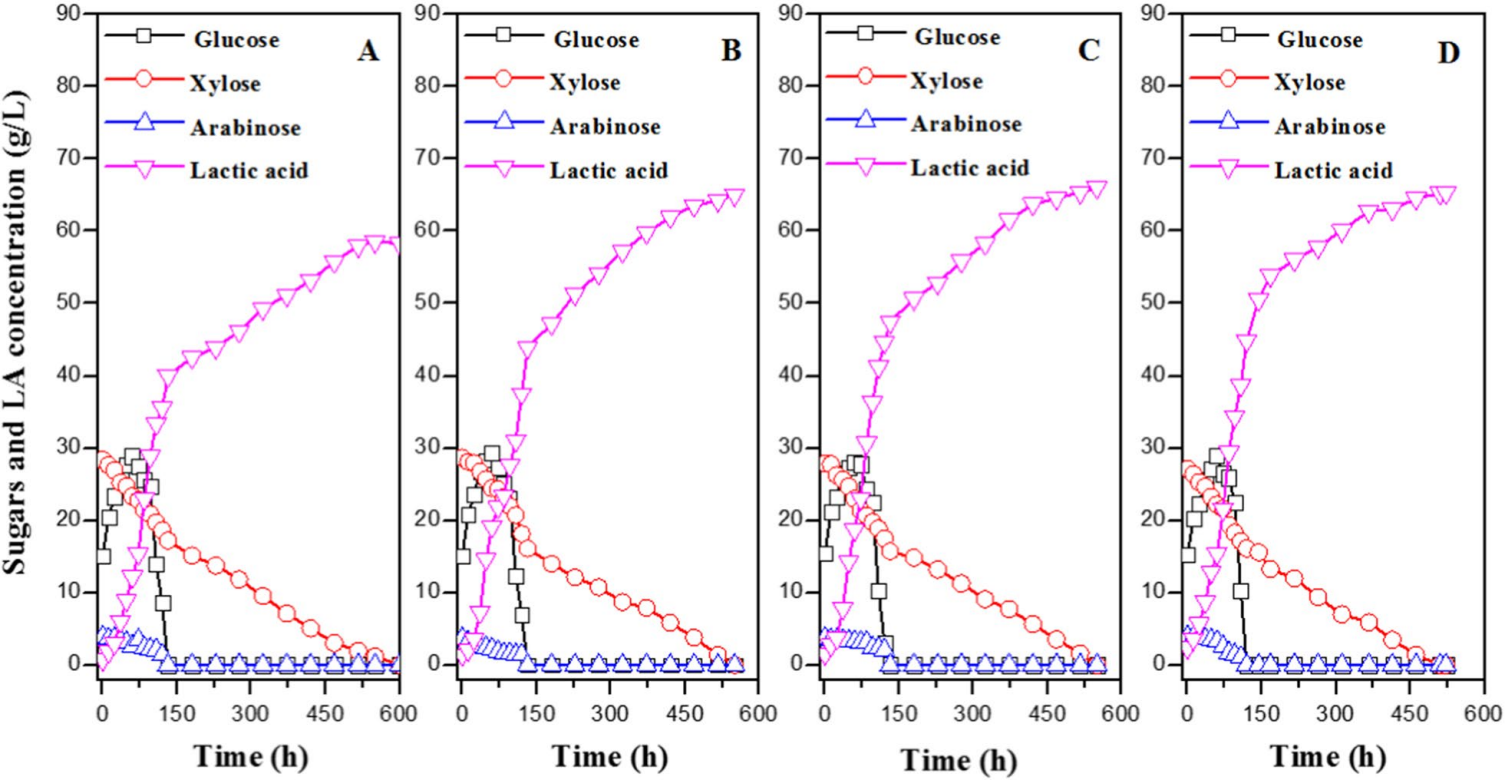
The effect of CSLP concentration on LA production via SSCF process from non-detoxified pre-CS. **A** 10 g/L CSLP. **B** 15 g/L CSLP. **C** 20 g/L CSLP. **D** 25 g/L CSLP. Conditions: 1 L bioreactor containing 500 mL CSLP medium, 20% (w/v) dry CS loading including 9.74 g/L total inhibitors, 35 FPU/g-CS cellulase, 12 h pre-hydrolysis time, 50 °C, pH 5.5

CSLP is an important nutrient for LA production, and it has been tested to improve LA productivity using sugarcane molasses, starchy biomass, etc. [14, 20, 21]. In this study, adding 10–20 g/L CSLP gradually increased LA concentration from 58.28 g/L to 66.11 g/L, while 25 g/L CSLP yielded 65.33 g/L-LA (Fig. 4). The rate of glucose and xylose consumption increased as the amount of CSLP added increased. As a result, the maximum productivity was obtained using 25 g/L CSLP. Given the economics of the SSCF process, 20 g/L CSLP was deemed an appropriate nitrogen source addition.

**Fig. 5:**
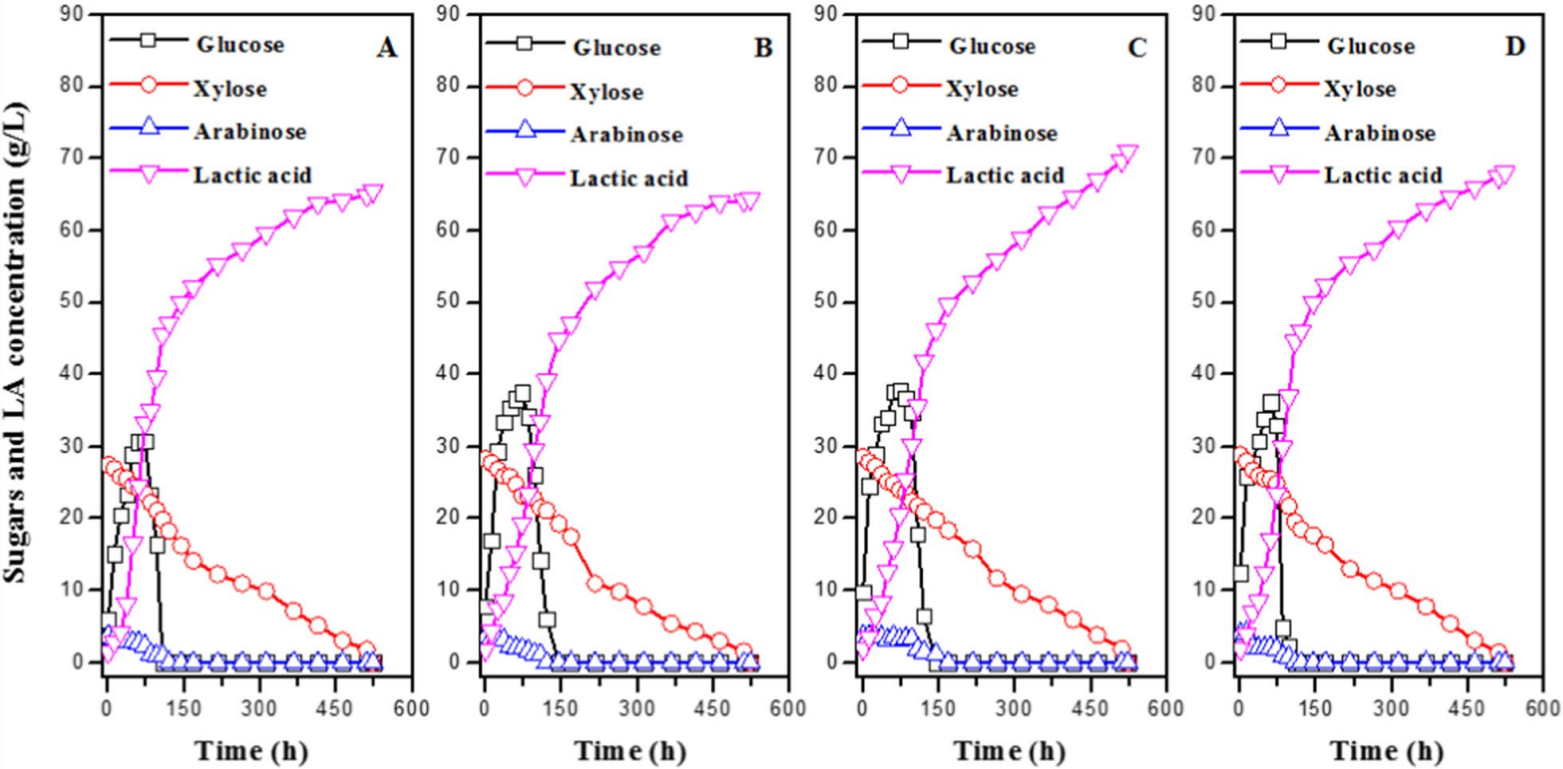
The effect of pre-hydrolysis time on LA production via the SSCF process from non-detoxified pre-CS. **A** 0 h, **B** 2 h, **C** 4 h, **D** 6 h. Conditions: 1 L bioreactor containing 500 mL CSLP medium, 20% (w/v) dry CS loading including 9.74 g/L total inhibitors, 35 FPU/g-CS cellulase, 20 g/L CSLP, 50 °C, pH 5.5

Pre-hydrolysis time was then investigated in the SSCF process by microbial consortium DUT50 (Fig. 5). The initial reducing sugars, including glucose, xylose, and arabinose, increased from 37.15 g/L to 45.46 g/L as the pre-hydrolysis time increased from 0 to 6 h. The highest glucose concentrations of 30.86 g/L, 37.50 g/L, 37.82 g/L, and 36.15 g/L were attained at 72 h, 72 h, 72 h, and 60 h when the pre-hydrolysis time periods were 0 h, 2 h, 4 h, and 6 h, respectively. The highest LA concentration of 71.04 g/L was achieved using 4 h pre-hydrolysis of pretreated CS.

The above results guided the feasibility of pre-CS utilization without detoxification in LA production by thermophilic microbial consortium DUT50. Furthermore, the optimum conditions for the SSCF process of pre-CS without the operations of solid–liquor separation and detoxification are 20% (w/v) dry biomass loading, 35 FPU/g-CS cellulase, 20 g/L CSLP, and 4 h of pre-hydrolysis time. As a result, 71.04 g/L-LA with a yield of 0.49 g/g-CS and a purity of L-LA of 96.8% were obtained. The comparatively higher LA concentrations of 101.9 g/L and 77.66 g/L were obtained from dry dilute acid pretreated and bio-detoxified CS by reported *P. acidilactici* DQ2 and Engineered *P. acidilactici* TY112, respectively [7, 9]. However, the lower corn stover yields of 0.38 g/g and 0.26 g/g were achieved because the xylose was not utilized by these two microorganisms. Due to the high tolerance of the microbial consortium DUT50 to inhibitors, a higher lactic acid yield to corn stover of 0.49 g/g was obtained in this study without detoxification. This is the highest lactic acid production reported from non-detoxified acid pretreated corn stover via the SSCF process without the generation of wastewater.

### The mechanism of microbial consortium for advanced performance

**Fig. 6:**
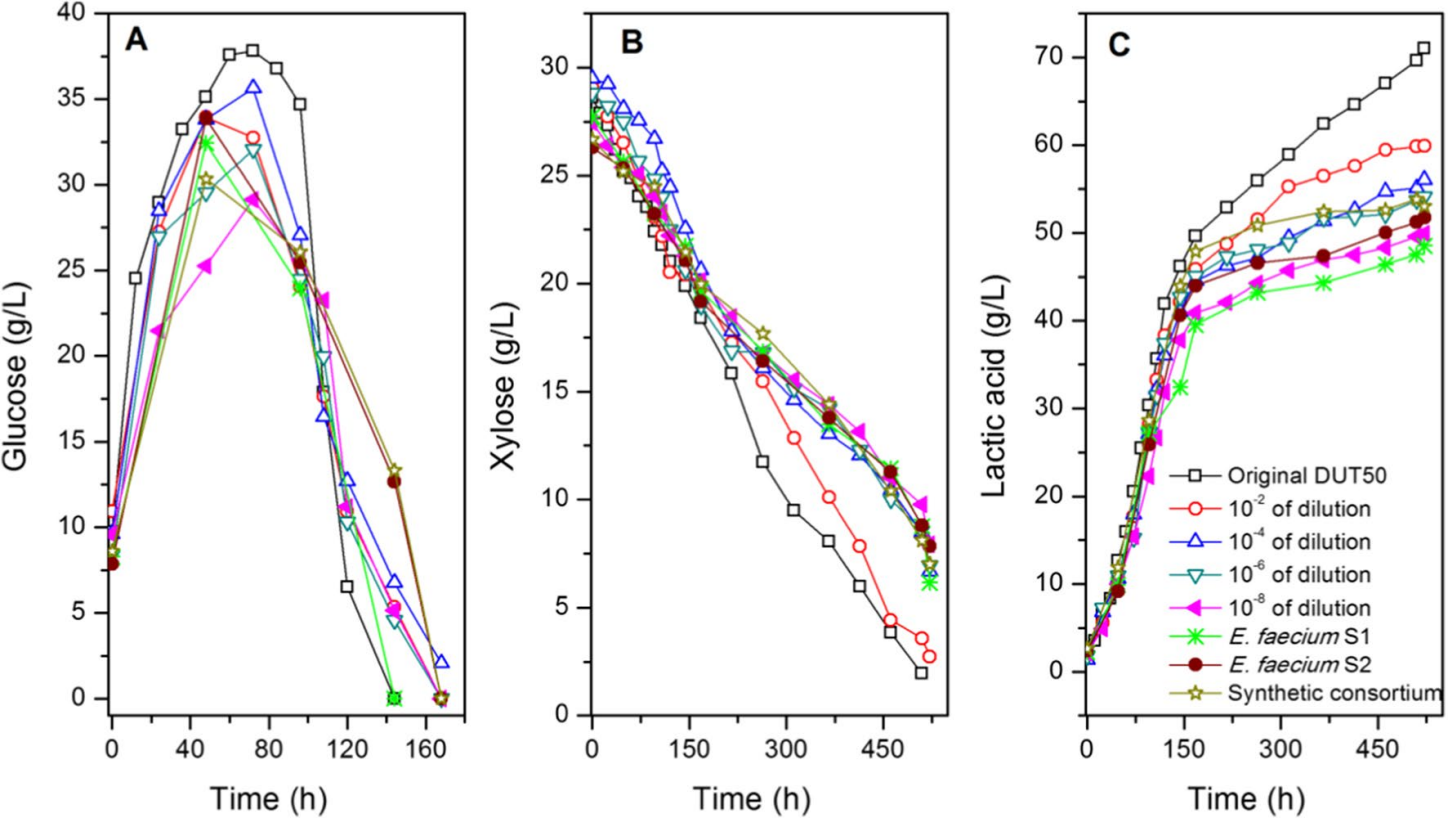
Performances of diluted consortia, *E. faecium*, and synthetic consortium for LA production from non-detoxified pre-CS. Conditions: 1 L bioreactor containing 500 mL CSLP medium, 20%(w/v) dry CS loading including 9.74 g/L total inhibitors, 35 FPU/g-CS cellulase, 20 g/L CSLP, 4 h pre-hydrolysis time, 50 °C, pH 5.5

Serial dilution is an easy and efficient way to isolate the microbial consortium with the simplest community structure and target function without isolating pure strains [22, 23]. As a result, in this study, the original microbial consortium DUT50 was serially diluted in sterile saline, and the performance of diluted consortia was evaluated, as shown in Fig. 6. The performance of diluted microbial consortia decreased as dilution increased from 10^–2^ to 10^–8^. The mini consortia consumed xylose slowly compared to the original microbial consortium DUT50. Bacterial community analysis revealed that *Lactobacillus*, *Bacillus*, *Lactococcus*, *and Trichococcus* were eliminated after 10^–2^ dilution, while the abundance of *Enterococcus* increased by more than 98.50%. The findings could be explained by the fact that these four genera use xylose efficiently. To verify this hypothesis, two strains of *E. faecium* DUT-S1 and DUT-S2 were isolated to utilize non-detoxified pre-CS to produce lactic acid, respectively. Xylose consumption rate was also decreased, with 6.15 and 7.84 g/L xylose remaining in medium for DUT-S1 and S2 after 522 h, resulting in low lactic acid concentration and productivity, respectively.

In addition, the synthetic consortium of *E. faecium* DUT-S1 and DUT-S2 were constructed to improve the utilization of non-detoxified acid pretreated CS. In the synthetic microbial consortium, the consumption rate of xylose was also observed to decrease. The synthetic consortium left about 7.01 g/L xylose in fermentation medium after 522 h. Finally, the synthetic consortium obtained 53.03 g/L-lactic acid with a yield of 0.36 g/g-CS. The fermentation performance of the synthetic consortium prior to the single strain implied that the interaction mechanism of *E. faecium* DUT-S1 and DUT-S2 collaborated for non-detoxified CS utilization. However, when compared to DUT50, the synthetic microbial consortium produced and yielded less lactic acid. As a result, the strains of the genus, such as *Lactobacillus*, *Bacillus*, *Lactococcus*, *and Trichococcus,* may play an important role in xylose utilization and LA production for the utilization of non-detoxified pre-CS. Among these low abundance genus, thermophilic *Bacillus* and *Lactobacillus* strains have recently demonstrated homo-fermentative behaviors by metabolizing glucose and xylose simultaneously [5, 6, 24]. Xylose could be homo-fermented to LA by *B. coagulans*. Furthermore, in the presence of glucose, *B. coagulans* NBRC12714 consumed xylose at an average rate of 1.35 g/(L h) [5]. *Lactobacillus pentosus* FL0421 was reported to metabolize xylose via the phosphoketolase pathway at high xylose concentration, and acetic acid was produced as byproduct [24]. Furthermore, *P. acidilactici* PA 204 with a high tolerance of temperature and high-efficiency utilization of xylose was investigated [10]. The average productivity of 1.28 g/(L h) for SSF fed-batch process was obtained at 12% (w/w) NaOH pretreated and washed stover feeding. Xylose utilization was also enhanced by constructing xylose-assimilating pathways in a d-lactic acid-producing *P. acidilactici* bacterium. The xylose assimilation was significantly accelerated after long-term adaptive evolution. As a result, the xylose conversion ratio of 92.6% was achieved by SSCF at 25% (w/w) solids content of dry dilute acid pretreated and bio-detoxified CS [25]. In this study, we have tried using an adaptive evolution engineering strategy to develop a microbial consortium for faster xylose consumption. A microbial consortium was enriched by increasing xylose concentration from 5 g/L to 15.87 g/L at a high inhibitors concentration of 6.99 g/L and 50 °C. However, its ability to utilize xylose was not improved efficiently at the high temperature and inhibitors concentration (data not shown). Further study will focus on accelerating xylose consumption by adding the strains or consortia utilizing xylose efficiently, such as engineered bacteria.

The results presented in this study indicated that the enriched and adapted microbial consortium DUT50 consisting of *Enterococcus*, *Lactobacillus*, *Bacillus*, *Lactococcus*, *and Trichococcus* is highly functional. Throughout the fermentation period, no other organic acid or ethanol was detected, indicating that the microbial consortium DUT50 is primarily composed of homo-fermentative lactic acid bacteria and that the pathway of xylose metabolism may be the pentose phosphate pathway. In this study, a lactic acid titer of 71.04 g/L was obtained, which is approximately 37% and 34% higher than that obtained by single cultures of *E. faecium*, and the synthetic microbial consortium consisting of *Enterococcus*, respectively.

## Conclusions

In this study, adaptive evolution engineering was used to enrich a thermophilic microbial consortium for LA production. The consortium could be resistant to inhibitors concentration up to 9.74 g/L, and simultaneously metabolizes hexose and pentose without CCR effect. A simplified and feasible process only consisting of CS pretreatment by dilute acid and SSCF process was developed for LA production. A LA titer of 71.04 g/L with a yield of 0.49 g/g-CS was achieved from 20% (w/v) dry CS loading. The interaction mode of *Enterococcus* in the consortium was collaboration, whereas the low abundance of *Lactobacillus* and *Bacillus* might metabolize xylose efficiently via the pentose phosphate pathway.

## Materials and methods

### Raw materials, enzymes, and medium

Corn stover (CS) containing 31.18 wt% of cellulose, 26.95 wt% of hemicellulose, and 16.43 wt% of lignin was harvested from a farmer in Shandong province of China. The commercial cellulase of Cellic^®^CTec2 used was purchased from Novozymes (China). CSLP with a nitrogen content of 9% (w/v) was afforded by Yuancheng Biotechnology Company, Liaoning, China [14]. All other chemicals were of reagent grade and commercially available.

The toxic enrichment medium (TEM) and seed medium were as follows: 6.99 g/L total inhibitors (1.75 g/L acetic acid, 1.82 g/L furfural, 1.49 g/L 5-HMF and 1.93 g/L vanillin), 35 g/L glucose, 3 g/L xylose, 16 g/L CSLP, 2 g/L ammonium citrate, 2 g/L sodium acetate, 2 g/L K_2_HPO_4_, 0.20 g/L MgSO_4_·7H_2_O, and 0.05 g/L MnSO_4_·H_2_O.

The fermentation medium used was as follows: 10–20% (w/v) dry CS loading, 10–25 g/L CSLP, 2 g/L ammonium citrate, 5 g/L sodium acetate, 2 g/L K_2_HPO_4_, 0.58 g/L MgSO_4_·7H_2_O, and 0.25 g/L MnSO_4_·H_2_O.

### Dilute sulfuric acid pretreatment of CS and non-detoxified SSCF process

CS was milled into particles with size < 2 mm and then pretreated by 1% (v/v) dilute H_2_SO_4_ solution with dry biomass loading of 10–25% (w/v). The pretreatment was carried out in a tank at 121 °C for 120 min, followed by cooling to room temperature and pH adjustment to 5.5 using KOH [26].

Following pretreatment, the contents, including hydrolysate liquor and residues, were used as the substrate for SSCF process to produce lactic acid. The SSCF process was performed under a non-sterilized condition in a 1 L bioreactor filled with 0.5 L of fermentation medium at 50 °C with the agitation of 200 rpm and inoculation size of 5% (v/v). Before inoculating the seed culture, 35 FPU/g-CS of cellulase was added into the unsterile medium. The pH was maintained at 5.5 by using an automatic feed of 2 mol/L NaOH solution. Samples were taken periodically to determine the concentration of products and reducing sugar.

### Adaptive evolution engineering of the microbial consortium to temperature and inhibitors

The adaptation protocol was established to select thermophilic and inhibitors-tolerant microbial consortia, thereby increasing the utilization of non-detoxified pre-CS. First, cattle stomach content was prepared by adding 10 g fresh content into 15 mL saline solution, mixing for 3 min with a vortex. Then, cattle stomach content was then inoculated with 5% incubation into a toxic enrichment medium using an adaptive strategy of increasing temperature (42 °C, 45 °C, 47 °C, 50 °C). After consuming half of the reducing sugars, the culture was transferred to a fresh adaptation medium with a higher temperature for subsequent cultivation. The culture was cultivated five times at the same temperature before exposure to the higher one. After 20 generations of long-term domestication with non-detoxified hydrolysate liquor of pre-CS, the stable thermophilic and inhibitors-tolerant microbial consortium DUT50 was achieved.

### The construction of mini consortia and isolation of single strain

Mini consortia were constructed by serial dilution (10^–2^ to 10^–8^) of the original consortium DUT50 with sterile saline and then incubated in the seed medium. Once sugars were depleted, the enriched consortia were serially transferred to the fresh seed medium with a 5% inoculation three times to ensure a stable microbial composition.

By streaking on a solid medium with bromocresol green as an indicator, single strains from consortium DUT50 were isolated and purified. As lactic acid production increased, the color of indicator gradually shifted from blue to green. Twelve single bacterial colonies with high lactic acid production concentrations were chosen based on the color of the indicator. Two strains with perfect utilization of CS hydrolysate were studied further out of a total of 12 strains. BLAST analysis of the 16S rRNA gene sequence of the isolated two strains demonstrated 100% similarity to *Enterococcus faecium* CBA7134. Thus, these two strains were designated as *E. faecium* DUT-S1 and *E. faecium* DUT-S2. The 16S rRNA sequence of *E. faecium* DUT-S1 and DUT-S2 was deposited in the GenBank database under accession numbers MW479185.

### Composition analysis of the microbial consortium

The bacterial community composition of microbial consortia was investigated by 16S rRNA gene amplicon high-throughput sequencing provided by Sangon Biotech in Shanghai, China. 16S rRNA gene sequences for the consortia DUT50 during the adaptive evolution process and dilution consortia have been submitted to the NCBI Sequence Read Archive, and the corresponding accession numbers are shown in Table 2.

### Analytical methods

Glucose, xylose, arabinose, and organic acid (lactic acid, acetic acid) were analyzed using high-performance liquid chromatography (HPLC) equipped with an Aminex HPX-87H column with a column temperature of 65 °C. Sulfuric acid (5 mmol/L) was the mobile phase with 0.6 mL/min flow rate. The major inhibitors (furfural, 5-HMF, and vanillin) in hydrolysate liquor of pre-CS were analyzed using HPLC equipped with a Supersil ODS2 column (Elite) and a UV detector at 285 nm [16]. The optical purity of the l-lactic acid was determined by HPLC equipped with an Astec^®^ CLC-L Chiral column (Sigma Aldrich, Co.) maintained at 25 °C with a UV detector at 254 nm [20].

The lactic acid yield (Y) was calculated by dividing the total lactic acid produced by the dry matter of CS, expressed in g/g. The lactic acid productivity was calculated by dividing lactic acid produced by the fermentation time, expressed in g/(L h).

## Data Availability

The datasets used and/or analyzed during the current study are available from the corresponding author on reasonable request.

## Abbreviations

LA: Lactic acid
CS: Corn stover
CSLP: Corn steep liquor power
SSCF: Simultaneous saccharification and co-fermentation
CCR: Carbon catabolite repression
Pre-CS: Acid pretreated corn stover
HMF: Hydroxymethylfurfural
